# Local Infiltration Analgesia Versus Adductor Canal Block for Managing Pain After Anterior Cruciate Ligament Reconstruction: A Systematic Review and Meta-analysis

**DOI:** 10.1177/23259671241292029

**Published:** 2024-11-12

**Authors:** Shaheer Nadeem, Reza Ojaghi, Partha Patel, Eric Locke, Andrew McGuire, Michael A. Pickell

**Affiliations:** * Division of Orthopaedic Surgery, University of Ottawa, Ottawa, Ontario, Canada; Investigation performed at the Division of Orthopaedic Surgery, University of Ottawa, Ottawa, Ontario, Canada

**Keywords:** ACL reconstruction, local infiltration analgesia, adductor canal block

## Abstract

**Background:**

Adductor canal block (ACB) and local infiltration analgesia (LIA) are frequently used to manage pain in patients after anterior cruciate ligament reconstruction (ACLR).

**Purpose:**

To compare the difference in pain scores and opioid consumption between ACB and LIA for ancillary pain management in patients after ACLR.

**Study Design:**

Systematic review; Level of evidence, 3.

**Methods:**

A literature search was conducted using PubMed, MEDLINE, and Embase databases according to PRISMA (Preferred Reporting Items for Systematic Reviews and Meta-Analyses) guidelines. Studies that compared pain scores at 2, 6, 12, or 24 hours after ACLR or provided information on 24-hour opioid consumption were included. Of 240 publications initially screened by abstract and title, 4 studies were included, and data related to participant characteristics, anesthetic technique, and pain-related outcomes were extracted. The standardized mean difference (MD) in pain scores and morphine milligram equivalents consumed in 24 hours was compared using a random-effects model.

**Results:**

In all studies, ropivacaine was the primary anesthetic used for LIA and ACB, with 1 study also employing bupivacaine as an alternative. The difference in pain scores between LIA and ACB was not significant at 2 hours (MD, 0.04 [95% CI, –0.22 to 0.29]; *P* = .79), 6 hours (MD, 0.16 [95% CI, –0.20 to 0.52]; *P* = .39), 12 hours (MD, 0.54 [95% CI, –0.49 to 1.56]; *P* = .31), or 24 hours (MD, 0.12 [95% CI, –0.10 to 0.34]; *P* = .28). The difference in morphine milligram equivalents was also not statistically significant (MD, –0.07 [95% CI, –0.25 to 0.11]; *P* = .68).

**Conclusion:**

From this review, the authors suggest considering LIA over ACB because of its potential to offer comparable pain relief and opioid consumption while being less time intensive. However, the study results should be interpreted with caution, given the limited number of studies included.

Anterior cruciate ligament reconstruction (ACLR) stands as one of the most frequently performed orthopaedic surgical procedures in the United States and Canada, with an estimated 200,000 ACL injuries and US$7 billion cost annually in the United States alone.^9,15,20^ It is well documented that effective pain management during the perioperative and postoperative periods is crucial not only for ensuring patient satisfaction but also for promoting early mobilization and successful rehabilitation.^4^ Additionally, effective perioperative analgesia is important because pain after ACLR is typically greatest in the first 24 hours after surgery.^25^

Regional anesthesia is a commonly used technique for perioperative pain control in ACLR.^2^ This form of anesthesia, also known as nerve block, is lauded for its ability to effectively manage pain while concurrently reducing the requirement for oral narcotic medications.^19^ Among the various regional blocks used for ACLR, 2 prevalent options are adductor canal block (ACB) and the femoral nerve block (FNB).^12,17^ FNB entails administering an anesthetic around the femoral nerve and is frequently applied in procedures involving the anterior thigh and knee.^12^ A study on FNB found that although it provides effective analgesia, it may lead to postoperative quadriceps weakness and reduced participation in early rehabilitation.^6^ Consequently, FNB has been found to be associated with a greater risk of postoperative falls compared to other regional anesthetic techniques.^24^ In contrast, ACB involves the local infiltration of an anesthetic into the adductor canal at the proximal thigh level. This results in blockade of the saphenous nerve and the nerve to the vastus medialis, offering anesthesia within the distribution of the saphenous nerve without causing motor blockade of the entire quadriceps group. Moreover, research suggests that ACB provides equivalent anesthesia to FNB while preserving quadriceps muscle strength.^1,17^

As an alternative to regional anesthesia, local infiltration analgesia (LIA) is frequently employed to deliver nonnarcotic pain relief after surgery.^21^ Traditionally, LIA entails injecting an anesthetic around the surgical site.^21^ This results in pain inhibition localized to the site of administration.^18^ LIA does not necessitate the use of ultrasound-guided imaging, as is typically required for regional blocks, thereby saving valuable perioperative time. Additionally, LIA eliminates the potential morbidity associated with nerve blocks, such as nerve injuries, and does not impact the quadriceps muscle, theoretically enhancing participation in early rehabilitation.^10^

While several studies have examined the analgesic effectiveness of ACB compared to LIA, to our knowledge, no systematic review or meta-analysis has been conducted. Therefore, given the increasing adoption of both LIA and ACB as alternatives to FNB because of concerns about quadriceps weakness,^6^ it is prudent to conduct a systematic comparison between these 2 methods. Based on research comparing LIA to other regional blocks,^1,17^ our hypothesis was that LIA and ACB would provide equivalent analgesia in patients undergoing ACLR.

## Methods

### Literature Search

This study adhered to the PRISMA (Preferred Reporting Items for Systematic Reviews and Meta-Analyses) guidelines.^14^ A comprehensive literature search was conducted between the inception of relevant databases and June 2023, including PubMed, MEDLINE, and Embase. Search terms included “ACL,”“anterior cruciate ligament,”“ACB,”“adductor canal block,”“saphenous nerve,”“LIA,”“local infiltration analgesia,”“intra-articular inject*,” and “peri-articular inject*.” These search terms were combined using standard Boolean operators. Furthermore, the references of retrieved articles were manually searched to identify any additional eligible studies.

Inclusion criteria encompassed studies that compared outcomes in patients who underwent ACLR using either LIA or ACB. Additionally, only studies reporting opioid consumption at the 24-hour postoperative mark or pain scores measured with a visual analog scale (VAS) or numeric rating scale (NRS) at 2, 6, 12, or 24 hours postoperatively were considered. The NRS is a linear scale that asks patients to rate their pain from 0 to 10, with 0 indicating no pain and 10 representing the worst imaginable pain. Similarly, the VAS asks patients to mark their pain on a 10-cm line, with extreme anchor points representing no pain (0 cm) or the worst imaginable pain (10 cm). Studies reporting results using the VAS or NRS were considered eligible for inclusion in the meta-analysis, given that both measures are linear and have similar ranges. Studies were excluded if they fell into the categories of reviews or case series or if they exclusively included pediatric patients (age, <18 years). Studies not published in English were also excluded.

### Article Screening

After completion of the literature search, duplicate articles were eliminated. There were 2 independent reviewers (S.N. and P.P.) who screened the titles and abstracts of all articles according to the eligibility criteria. Subsequently, the full texts of the remaining studies were subjected to further scrutiny. In instances of disagreements between reviewers, conflicts were resolved through a discussion.

**Figure 1.**
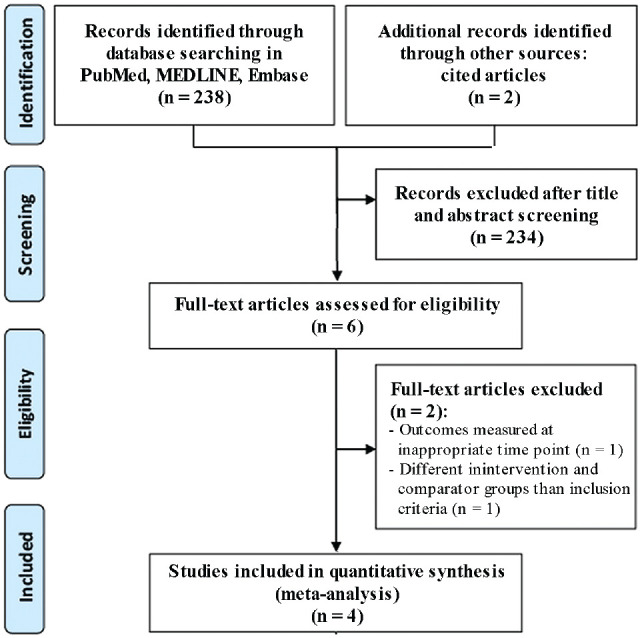
PRISMA (Preferred Reporting Items for Systematic Reviews and Meta-Analyses) flowchart of the article selection process.

After the retrieval of search results and the removal of duplicates, a total of 238 studies were identified. Additionally, 2 studies were added after reviewing the reference lists of other included articles. In total, the titles and abstracts of 240 articles were evaluated for eligibility. The full text of 6 articles was evaluated. One study was excluded because it compared LIA against FNB instead of ACB.^3^ Another study was excluded because it did not provide 24-hour opioid consumption data or pain scores at 2, 6, 12, or 24 hours.^7^ After conducting a full-text screening, 4 studies^2,13,19,23^ met the inclusion criteria for this review. The flowchart of the screening process is shown in Figure 1.

### Data Extraction

The same 2 reviewers (S.N. and P.P.) autonomously extracted data from the included articles, including study characteristics, participant characteristics, graft type, intervention, and outcomes. All collected variables were recorded in a password-protected spreadsheet. In the event of discrepancies during data extraction, a third reviewer (R.O.) was consulted.

### Risk-of-Bias Assessment

The methodological quality of each study was independently evaluated by 2 authors (S.N. and R.O.). The revised version of the Cochrane risk-of-bias tool was employed for assessing the quality of randomized controlled trials (RCTs).^8^ For observational and case-control studies, methodological quality was assessed using the Methodological Index for Non-Randomized Studies (MINORS).^22^

### Statistical Analysis

The primary outcome assessed in this study involved the mean difference (MD) in VAS or NRS pain scores at 2, 6, 12, and 24 hours postoperatively between patients administered LIA and those administered ACB. As a secondary outcome, the MD in opioid consumption at 24 hours postoperatively between the 2 study groups was also analyzed. Studies reporting side effects were subjected to a comparative analysis of any differences between the groups. This study did not differentiate between periarticular and intra-articular injections; hence, no subgroup analysis was conducted. A meta-analysis was considered feasible if data from ≥2 studies were available.

All statistical analyses were performed using Review Manager (Version 5.4; The Cochrane Collaboration). The 95% CI was applied, and results were deemed statistically significant if *P*≤ .05. A random-effects model was employed to calculate the standardized MD between the study groups. In cases in which the standard deviation was not reported, it was estimated using Review Manager. Heterogeneity was assessed using the *I*^2^ statistic. To standardize data on opioid consumption, morphine milligram equivalents (MMEs) at 24 hours were calculated using equivalence values provided by the US Centers for Disease Control and Prevention.^5^

## Results

### Characteristics of Included Studies

Of the 4 included studies, 3 studies^2,13,23^ were RCTs, and 1 study^19^ was a retrospective chart review. All studies were conducted at single institutions, with 2 taking place in India,^2,13^ 1 in the United States,^19^ and 1 in Switzerland.^23^ The publication dates ranged from 2019 to 2022, and a total of 406 patients were included in the review. The majority of the studies used autografts, with the semitendinosus-gracilis and bone–patellar tendon–bone grafts being the most common. The method of LIA varied: an intra-articular injection was used in 2 studies,^2,13^ a periarticular injection in 1 study,^23^ and a combination of periarticular and intra-articular injections in 1 study.^19^ Table 1 provides a comprehensive overview of the study characteristics.

**Table 1:** Characteristics of Included Studies^*a*^

First Author (Year)	Study Design	Anesthetic Type	No. of Knees (Male)	Age,^*b*^ y	Graft Type and Concomitant Procedures	Method of LIA
Stebler^23^ (2019)	RCT	• LIA: 20 mL of 0.5% ropivacaine • ACB: 20 mL of 0.5% ropivacaine	• LIA: 49 (38) • ACB: 49 (32)	• LIA: 28 ± 8.8 • ACB: 29 ± 6.7	ST-G autograft	Periarticular infiltration of graft site, iliotibial band, and subcutaneous tissue
Schaver^19^ (2022)	Retrospective chart review	• LIA: 300 mg of 0.5% ropivacaine, 30 mg of 30 mg/mL ketorolac, 0.6 mg of 1 mg/mL epinephrine, and 5 mg of 0.5 mg/mL morphine mixed with 100 mL of 0.9% sodium chloride • ACB: 15-20 mL of 0.5% bupivacaine or 0.5% ropivacaine	• LIA: 157 (80) • ACB: 108 (54)	• LIA: 23.4 ± 20.6 • ACB: 27.5 ± 96	BPTB autograft, hamstring tendon autograft, quadriceps tendon autograft, or tibialis anterior allograft	Periarticular infiltration of subcutaneous tissue and intra-articular injection
Bangal^2^ (2020)	RCT	• LIA: 20 mL of 0.2% ropivacaine • ACB: 20 mL of 0.2% ropivacaine	• LIA: 30 (22) • ACB: 30 (21)	• LIA: 30.6 (15-50) • ACB: 30.8 (15-50)	BPTB or ST-G autograft	Intra-articular injection
Mittal^13^ (2021)	RCT	• LIA: 9 mL of 0.2% ropivacaine and 1 mL of 150 μg clonidine • ACB: 9 mL of 0.2% ropivacaine and 1 mL of 150 μg clonidine	• LIA: 30 (17) • ACB: 30 (21)	• LIA: 38.2 ± 11.3 • ACB: 31.9 ± 9.6	NR	Intra-articular injection

a ACB, adductor canal block; BPTB, bone–patellar tendon–bone; LIA, local infiltration analgesia; NR, not reported; RCT, randomized controlled trial; ST-G, semitendinosus-gracilis tendon.

b Data are reported as mean ± SD or mean (range).

### Methodological Quality

There were concerns regarding the risk of bias in all RCTs assessed in this study. There were some concerns regarding randomization, deviation of interventions, measurement of the outcome, and selection of reported results in the study by Bangal and Ghodki.^2^ In the study by Stebler et al,^23^ there was some concern regarding the selection of reported results because no protocol was provided. Similarly, there were some concerns in the study by Mittal et al^13^ for the deviation of interventions and selection of reported results. Only the study by Schaver et al^19^ was evaluated using the MINORS, receiving a score of 17 of a possible 24 (fair). There was a degree of a risk of bias associated with a lack of blinding, insufficient sample size calculation, and high ratio of loss to follow-up, among other factors.

### Pain Scores

**Figure 2.**
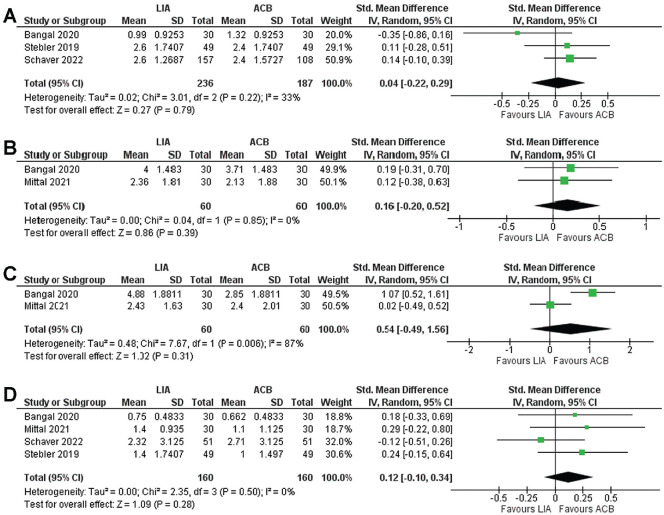
Forest plot of visual analog scale (VAS) or numeric rating scale (NRS) pain scores at (A) 2 hours (including data from 3 studies^2,19,23^), (B) 6 hours (including data from 2 studies^2,13^), (C) 12 hours (including data from 2 studies^2,13^), and (D) 24 hours (including data from all 4 studies^2,13,19,23^) after anterior cruciate ligament reconstruction. ACB, adductor canal block; IV, inverse variance; LIA, local infiltration analgesia; Std, Standard.

The VAS or NRS pain scores were compared between the LIA and ACB groups at 2, 6, 12, and 24 hours. Analysis at 2 hours postoperatively incorporated 3 studies,^2,19,23^ comprising 236 knees in the LIA group and 187 knees in the ACB group. The results indicated no significant difference in pain scores between the groups (MD, 0.04 [95% CI, –0.22 to 0.29]; *P* = .79) (Figure 2A).

At 6 and 12 hours, 2 studies^2,13^ were eligible for analysis, with a pooled sample size of 60 knees in each arm. The combined results showed no statistically significant difference in pain scores at 6 hours (MD, 0.16 [95% CI, –0.20 to 0.52]; *P* = .39) (Figure 2B) or 12 hours (MD, 0.54 [95% CI, –0.49 to 1.56]; *P* = .31) (Figure 2C). At 24 hours postoperatively, all 4 studies^2,13,19,23^ were analyzed, involving 160 patients in each arm. The collective results also revealed no significant difference between LIA and ACB (MD, 0.12 [95% CI, –0.10 to 0.34]; *P* = .28) (Figure 2D).

### Opioid Consumption

**Figure 3.**
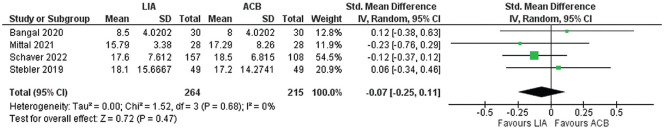
Forest plot of opioid consumption (morphine milligram equivalents) at 24 hours in all 4 studies.^2,13,19,23^ ACB, adductor canal block; IV, inverse variance; LIA, local infiltration analgesia; Std, Standard.

All 4 studies^2,13,19,23^ reported on opioid consumption at the 24-hour mark. After standardizing the dosages to MMEs, the pooled results were found to demonstrate no difference between the groups (MD, –0.07 [95% CI, –0.25 to 0.11]; *P* = .47) (Figure 3).

### Treatment-Related Side Effects

The insufficient reporting of treatment-related side effects in most studies precluded the possibility of conducting a meta-analysis. Mittal et al^13^ did not report the incidence of complications but did assess opioid-related side effects, finding no statistically significant difference between the 2 groups (*P* = .72). Similarly, Stebler et al^23^ found no instances of complications in either group. Additionally, these authors found no statistically significant difference in postoperative nausea and vomiting in patients receiving LIA versus ACB (2 hours: 2/47 vs 1/48, respectively [*P* = .46]; 24 hours: 10/39 vs 11/38, respectively [*P* = .46]; and 48 hours: 5/44 vs 4/45, respectively [*P* = .21]).^23^ Overall, among the studies providing data, no significant disparities in treatment-related side effects were observed in patients receiving ACB or LIA.

## Discussion

Overall, the findings of our review demonstrated that pain scores assessed with a VAS or NRS at 2, 6, 12, and 24 hours postoperatively, as well as MMEs, were similar between the intervention groups. It is important to note that while both treatment methods may offer similar analgesic effects, the consideration of functional outcomes is also crucial. However, because of the limited number of studies examining functional outcome variables, a meta-analysis of functional outcomes could not be conducted. Stebler et al^23^ reported a statistically significant difference in agility and Anterior Cruciate Ligament–Return to Sport after Injury scale scores at 8 months after ACLR, favoring the LIA group. However, this difference could potentially be attributed to errors, as the study was not adequately powered to assess these outcomes, and similar results were not observed at other time points. Importantly, these authors also found no significant difference in quadriceps muscle strength, range of motion, or walking distance between LIA and ACB at 24 hours, 48 hours, 4 months, and 8 months after ACLR. One of the primary reasons for choosing ACB over other regional blocks such as FNB is the preservation of quadriceps motor function.^1^ Because the current review found that LIA and ACB had similar analgesic effects and considering previous research on the safety of LIA on quadriceps function, these findings may further support the use of LIA, given its ease of use and avoidance of the minimal potential risks associated with nerve blocks.

Given the growing pressure on the health care system to manage increasing health care costs, resource stewardship must be a factor in treatment decision-making. Therefore, in addition to pain control and functional outcome scores, the time and cost differences between LIA and ACB should be considered. Administering ACB typically requires additional resources such as ultrasound guidance, anesthesia assistants, and dedicated procedure/block rooms.^17^ Especially in community settings, these essential resources may not always be readily available, potentially disrupting perioperative flow and leading to surgical delays. In contrast, LIA can be efficiently administered by directly infiltrating analgesia without significantly impacting operative time or perioperative patient flow.^18^ Additionally, ACB carries an increased risk profile compared to LIA, with potential complications including vascular injuries, intravascular infiltration, myositis, and nerve injuries, among others.^11,16^ Therefore, if both ACB and LIA provide similar levels of analgesia, LIA may be the preferable choice in ACLR.

### Strengths and Limitations

This systematic review and meta-analysis has several strengths that bolster the validity of our findings. The study was conducted in adherence to PRISMA guidelines,^14^ ensuring standardized and robust methods. Additionally, our review included the exploration of multiple medical databases and supplemented the literature search by manually examining the reference lists of included studies.

However, this study also has limitations, mainly stemming from the small number of included studies and the diversity in the methods used across the studies. Notably, most studies differed in the selection, concentration, and volume of anesthetic agents, which could result in heterogeneity. Furthermore, only 4 studies were included in our systematic review and meta-analysis, thus limiting its generalizability and raising the potential for bias in the results. Despite most of the included studies being RCTs, there were concerns regarding the risk of bias. Additionally, the sample size at some of the analyzed time points was small, with only 60 knees in each arm for the pooled effect estimate at 6 and 12 hours. The study did not differentiate between periarticular and intra-articular injections in terms of LIA, which could be explored in future research. Furthermore, analyzing side effects as part of a meta-analysis was not feasible, as many studies either lacked information on the topic or did not provide qualitative data. Despite these limitations, our study contributes meaningful data for clinicians and underscores the importance of further research on the subject.

## Conclusion

From this review, we suggest considering LIA over ACB because of its potential to offer comparable pain relief and opioid consumption while being less time intensive. However, the results should be interpreted with caution, given the limited number of studies included. Further investigation through large RCTs with standardized outcomes is essential to assess comparative functional outcomes.
